# The Role of Cancer Stem Cells in Drug Resistance in Gastroesophageal Junction Adenocarcinoma

**DOI:** 10.3389/fmolb.2021.600373

**Published:** 2021-02-08

**Authors:** Kate Dinneen, Anne-Marie Baird, Ciara Ryan, Orla Sheils

**Affiliations:** ^1^ School of Medicine, Trinity Translational Medicine Institute, Trinity College Dublin, Dublin, Ireland; ^2^ Department of Histopathology, St. James’s Hospital, Dublin, Ireland

**Keywords:** cancer stem cells, drug resistance, gastroesophageal junction adenocarcinoma, epithelial mesenchymal transition, microRNA

## Abstract

Gastroesophageal junction adenocarcinomas (GEJA) have dramatically increased in incidence in the western world since the mid-20^th^ century. Their prognosis is poor, and conventional anti-cancer therapies do not significantly improve survival outcomes. These tumours are comprised of a heterogenous population of both cancer stem cells (CSC) and non-CSCs, with the former playing a crucial role in tumorigenesis, metastasis and importantly drug resistance. Due to the ability of CSCs to self-replicate indefinitely, their resistance to anti-cancer therapies poses a significant barrier to effective treatment of GEJA. Ongoing drug development programmes aim to target and eradicate CSCs, however their characterisation and thus identification is difficult. CSC regulation is complex, involving an array of signalling pathways, which are in turn influenced by a number of entities including epithelial mesenchymal transition (EMT), microRNAs (miRNAs), the tumour microenvironment and epigenetic modifications. Identification of CSCs commonly relies on the expression of specific cell surface markers, yet these markers vary between different malignancies and indeed are often co-expressed in non-neoplastic tissues. Development of targeted drug therapies against CSCs thus requires an understanding of disease-specific CSC markers and regulatory mechanisms. This review details the current knowledge regarding CSCs in GEJA, with particular emphasis on their role in drug resistance.

## Introduction

Gastroesophageal junction adenocarcinomas (GEJA) are cancers which straddle the junction between the oesophagus and stomach, sharing similar epidemiological characteristics and risk factors to oesophageal adenocarcinoma (OAC) (Bray et al., 2018). Globally, these cancers have an average 5-year survival rate of 19.9% (Seer, 2019). Their incidence has increased by approximately 600% since the 1970s, with the majority of cases occurring in the Western world (Rubenstein and Shaheen, 2015). This epidemiological shift can be partially accounted for by changes in Western lifestyle including diet, increased rates of obesity, smoking and gastro-oesophageal reflux disease; however, the precise cause remains unclear (Buas and Vaughan, 2013). Despite early advances in treatment modalities, rates of disease recurrence and resistance to anti-cancer therapies remain high (Brungs et al., 2019), highlighting the need for further research into the epidemiology, management, molecular biology and classification of these tumours. This review focuses on drug therapies in GEJA, with a specific emphasis on the role of cancer stem cells (CSC) in the development of drug resistance and their potential utility as targets for novel anti-cancer therapies in GEJA.

### The Argument for GEJA as a Distinct Entity

Much of our current knowledge about GEJA is inferred from studies conducted on oesophageal and gastric adenocarcinomas. Malignancies of the gastroesophageal junction (GEJ) have traditionally been subdivided into cancers of either gastric or oesophageal origin. Clinical classification is based on the Siewert scoring system, which categorises tumours into three groups according to the location of their epicentre in relation to the gastric cardia: the epicentre of Siewert I tumours are 1-5 cm above; Siewert II tumour epicentres lie between 1 cm above and 2 cm below, and the epicentre of Siewert III tumours lies 2-5 cm below the gastric cardia (Siewert and Stein, 1998). By contrast, the TNM staging system is used to determine pathological classification. Whilst the 7^th^ edition of the TNM staged all GEJ tumours as oesophageal cancers, the 8^th^ edition was revised to treat Siewert III tumours as gastric cancers, similar to the original definition (Rice et al., 2017; Zanoni et al., 2018). However, many now believe that GEJ malignancies are best regarded as a separate disease entity with a distinct genetic signature, which could facilitate more accurate classification through a “cell of origin” model in the future (Hayakawa et al., 2016; Rice et al., 2017; Abdi et al., 2019; Lin et al., 2019). Indeed, the pathogenesis of OAC (Siewert I-II) and intestinal type adenocarcinoma of the gastric cardia (Siewert III) both arise in the setting of intestinal metaplasia, indicating potential shared carcinogenic pathways between the two anatomical locations. This pathological link is further supported by genetic studies, which suggest that metaplastic cells in Barrett’s oesophagus originate not from squamous progenitor cells, but rather from gastric cardia progenitor cells that have migrated to the lower oesophagus (Paulson et al., 2006; Quante et al., 2012). Additionally, recent genetic profiling studies demonstrated genetic similarities between chromosomal unstable subtype (CIN) gastric cardia adenocarcinomas and oesophageal adenocarcinoma of the GEJ (Bass et al., 2014; Kim et al., 2017).

### Current Treatment Options

Current treatment options for GEJA depend on the disease stage at diagnosis. Locally advanced non-metastatic GEJA is treated with a multimodal approach, usually a combination of surgical resection with neoadjuvant, perioperative and/or adjuvant chemotherapy, with or without concomitant radiotherapy (Lin et al., 2019). In early stage disease (Tis, T1a), minimally invasive approaches using endoscopic mucosal or submucosal resections may be possible, whilst frankly invasive tumours (T1b-4) require surgical resection. Surgery alone has unacceptably high rates of treatment failure, often due to advanced stage at presentation, thus most patients receive additional neoadjuvant or perioperative therapy such as Fluorouracil and Cisplatin. Several trial studies have examined these treatment options in lower oesophageal and gastric adenocarcinomas, both alone and in combination with surgery, from which data relating to GEJA has been extrapolated (Table 1). Whilst each showed a modest improvement in survival outcomes, the rates of overall survival (OS) and complete pathologic response (CPR) remained poor (Al-Batran et al., 2016). Approximately 55-60% of patients with early stage disease who undergo primary resection with curative intent will relapse within 5 years, and the median OS for patients with metastatic/recurrent disease is 11-12 months (Joshi et al., 2018). The poor response to these conventional therapies highlights a need for the development of more effective targeted therapies for both early and advanced stage disease.

**TABLE 1:** Completed Trial Outcomes for Current GEJA Treatments.

Study Name and Design	Survival Data	Clinical Trial Number
Neoadjuvant chemoradiotherapy plus surgery *vs.* surgery alone for oesophageal or junctional cancer (CROSS) (Shapiro et al., 2015).	Median OS 43.2 months *vs*. 27.1 months for surgery alone	Netherlands trial register number NTR487
Perioperative Epirubicin, Cisplatin, and infused Fluorouracil *vs.* surgery alone for incurable gastric, lower oesophageal or GEJ cancer (MAGIC) (Cunningham et al., 2006).	5-year survival 36% *vs*. 23% for surgery alone	Current controlled trials number ISRCTN93793971
Perioperative Fluorouracil + Cisplatin in resectable GEJA (ACCORD) (Ychou et al., 2011).	5-year survival 38% *vs*. 24% for surgery alone	Clinical trials gov number NCT00002883
Perioperative chemotherapy with Fluorouracil + Leucovorin, Oxaliplatin, and Docetaxel *vs.* ECF or ECX for resectable gastric or GEJ adenocarcinoma (FLOT4) (Al-Batran et al., 2019).	Median OS 50 months *vs*. 35 months in control ECF/ECX group	Clinical trials gov number NCT01216644

Abbreviations: OS, overall survival; ECF, Epirubicin, Cisplatin, and Fluorouracil; ECX, Epirubicin, Cisplatin, Capecitabine.

Early advances in our understanding of the molecular biology of GEJA have identified potential new treatment targets (Maron and Catenacci, 2017). Molecularly defined GEJA subsets have been observed that may hold therapeutic relevance, including tumours related to Epstein-Barr Virus; tumours with hyper-mutation, in particular microsatellite instable tumours; and those with homologous recombination deficiency (Janjigian et al., 2018). Many GEJAs are of CIN subtype, with amplifications in a range of receptor tyrosine kinases (RTKs), including EGFR and ERBB2 (Bass et al., 2014; Cristescu et al., 2015; Secrier et al., 2016; Kim et al., 2017). An additional class of drug which shows promise in GEJA are immune checkpoint inhibitors (ICIs), which help the immune system to attack cancer cells. Immunotherapeutic agents such as Pembrolizumab have been approved for use in chemotherapy refractory GEJA (Le et al., 2015; Muro et al., 2016; Janjigian et al., 2018; Greally et al., 2019). Ongoing trials are focusing on combinations of ICIs with established adjunct therapies, in addition to investigating the utility of novel drugs such as Ramucirumab–a vascular endothelial growth factor receptor 2 (VEGFR2) antagonist (Table 2). Whilst these trials have shown modest therapeutic benefits, the survival advantage for the patient nevertheless remains low. The fact that most trials focus on patients with disease refractory to first line therapies emphasises the ongoing issue of complex resistance mechanisms which circumvent anti-cancer drug treatments.

**TABLE 2:** Ongoing Phase 3 Trials of Targeted Therapies in GEJA.

Clinical Trials Identifier Number	Phase	Line	Disease Types	Intervention	Primary Endpoints
NCT02370498	III	II	Gastric and **GEJ** adenocarcinoma	Pembrolizumab *vs.* Paclitaxel in patients who progressed after therapy with Platinum and Fluoropyrimidine	PFS and OS
NCT03019588	III	II	Advanced gastric and **GEJ** adenocarcinoma	Pembrolizumab *vs.* Paclitaxel in patients who progressed after therapy with Platinum and Fluoropyrimidine	PFS and OS
NCT02314117	III	I	Metastatic gastric and **GEJ** adenocarcinoma	Capecitabine and Cisplatin +/- Ramucirumab	PFS
NCT02494583	III	I	Advanced gastric and **GEJ** adenocarcinoma	Pembrolizumab *vs.* Pembrolizumab + 5-FU or Capecitabine *vs.* Placebo + 5-FU or Capecitabine	PFS and OS
NCT01196390	III	I	Oesophageal and **GEJ** adenocarcinoma	Radiation therapy, Paclitaxel and Carboplatin +/- Trastuzumab	DFS
NCT02625610	III	I	Unresectable gastric and **GEJ** adenocarcinoma	Avelumab *vs.* continuation of first line chemotherapy	OS
NCT02581462	III	I	Gastric and **GEJ** adenocarcinoma	FLOT *vs*. FLOT + Herceptin/Pertuzumab	PFS and CPR
NCT02564263	III	II	Advanced oesophageal and **GEJ** adenocarcinoma	Pembrolizumab *vs.* investigator's choice standard therapy	OS
NCT02661971	III	I	Gastric and **GEJ** adenocarcinoma	FLOT *vs.* FLOT/Ramucirumab	OS and CPR
NCT03221426	III	1	Localized gastric and **GEJ** adenocarcinoma	Pembrolizumab + Chemotherapy (FP or XP) *vs.* Placebo + Chemotherapy (FP or XP)	OS, EFS and CPR
NCT02743494	III	II	Oesophageal and **GEJ** adenocarcinoma	Nivolumab *vs.* Placebo	DFS and OS

Abbreviations: GEJ, gastroesophageal junction; FP, 5-fluorouracil; XP, Cisplatin plus Capecitabine; EFS, event free survival; DFS, disease free survival; PFS, progression free survival; CPR, complete pathologic response

### Mechanisms of Treatment Resistance

**FIGURE 1:**
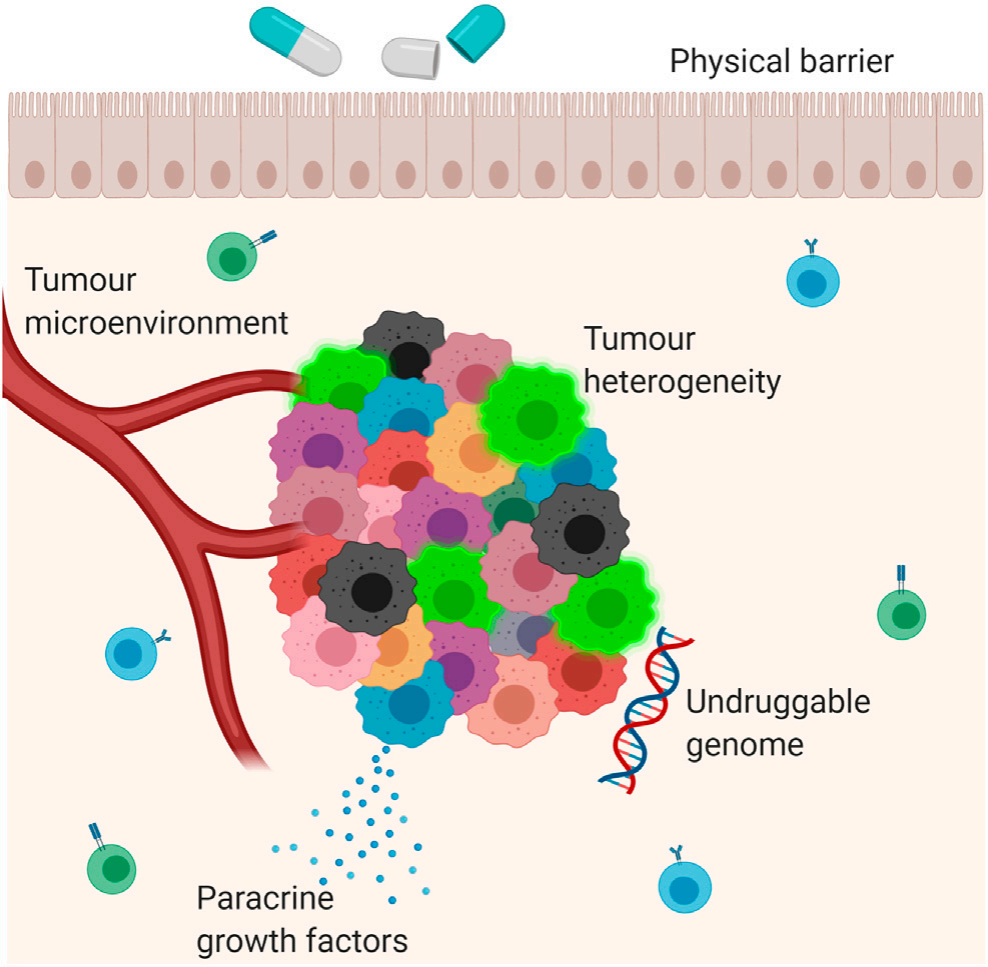
**Mechanisms of drug resistance.** Multiple different mechanisms contribute to the development of drug resistance in cancer. These include the interaction between the tumour and its microenvironment; secretion of paracrine growth factors which promote tumour growth; tumour heterogeneity; physical barriers and the ‘undruggable genome’, which refers to mutations which have not yet been targeted by anti-cancer therapies. The blue cells in the background are B lymphocytes. The green cells in the background are T lymphocytes. The bright green cells in the tumour mass are cancer stem cells.

Resistance to anti-cancer therapies persists as an obstacle to optimal clinical management and prognostication in GEJA. The mechanisms leading to drug resistance are complex and multifactorial, and the pharmacological impact of a particular therapeutic agent depends on both intrinsic and acquired tumour cell characteristics (Vasan et al., 2019), (Figure 1). For example, the interplay between the tumour and its microenvironment–that being the surrounding immune cells, stroma and vasculature–may mediate resistance through obstruction of drug absorption by the tumour cells or by stimulation of paracrine growth factors that promote tumour cell growth (Prieto-Vila et al., 2017; Vasan et al., 2019). Physical barriers include “sanctuary sites”, which are anatomical sites within which systemic therapies do not reach therapeutic concentrations (Toyokawa et al., 2015). The central nervous system is the main sanctuary site in the human body, with the blood brain barrier acting as a physical barrier; however, sanctuary sites may also exist at tissue level due to uneven drug distribution between different tissue types. Furthermore, across many different types of cancers there exists a number of oncogenes and tumour suppressor genes, many of which have yet to be targeted by anti-cancer therapies, including TP53 and MYC: the presence of this “undruggable genome” further contributes to tumour cell heterogeneity and hence drug resistance (Vasan et al., 2019).

Most tumours are comprised of a phenotypically diverse population of cancer cells, driven by a complex array of genetic and phenotypic alterations that disrupt normal cell cycle and cellular processes at multiple levels, including genomic, transcriptomic and influences from the tumour microenvironment (Prasetyanti and Medema, 2017; Shibue and Weinberg, 2017; Sharma et al., 2019; Tripathi et al., 2020). This diversity is known as intra-tumour heterogeneity and is thought to play a crucial role in the development of treatment resistance (Prasetyanti and Medema, 2017). Putative personalised therapies often fail because a single biopsy may sample only one sub-population of tumour cells, thus underestimating the heterogeneity present within a tumour (Gerlinger et al., 2012). Contributing to this complex heterogeneity is the presence of cancer stem cells (CSCs). This tumour cell population is of critical clinical importance and is known to contribute to resistance to anti-cancer therapies in many solid organ malignancies (Li and Li, 2014;Nunes et al., 2018).

### Cancer Stem Cells

CSCs are a small but crucially important sub-population of tumour cells which drive tumorigenesis, metastasis and treatment resistance (Prasetyanti and Medema, 2017). They are undifferentiated and capable of limitless self-renewal, with potential for subsequent differentiation into various non-CSC cell types which lack capacity for self-renewal or migration and instead form the bulk of the tumour (Reya et al., 2001). They were first identified in the 1990s when CD34^+^, CD38^−^ leukemic cells were shown to have bone marrow hematopoietic stem cell characteristics (Lapidot et al., 1994; Bonnet and Dick, 1997). In the 2003 seminal paper, Al-Hajj *et al* identified CSCs in solid tumours by demonstrating tumorigenic (stem) cells with cell surface marker profile CD44^+^, CD24^−/low^ in breast cancer (Al-Hajj et al., 2003). Shortly after, CSC markers were identified for other malignancies including prostate, colon, liver and lung (Medema, 2013; Eun et al., 2017).

**FIGURE 2:**
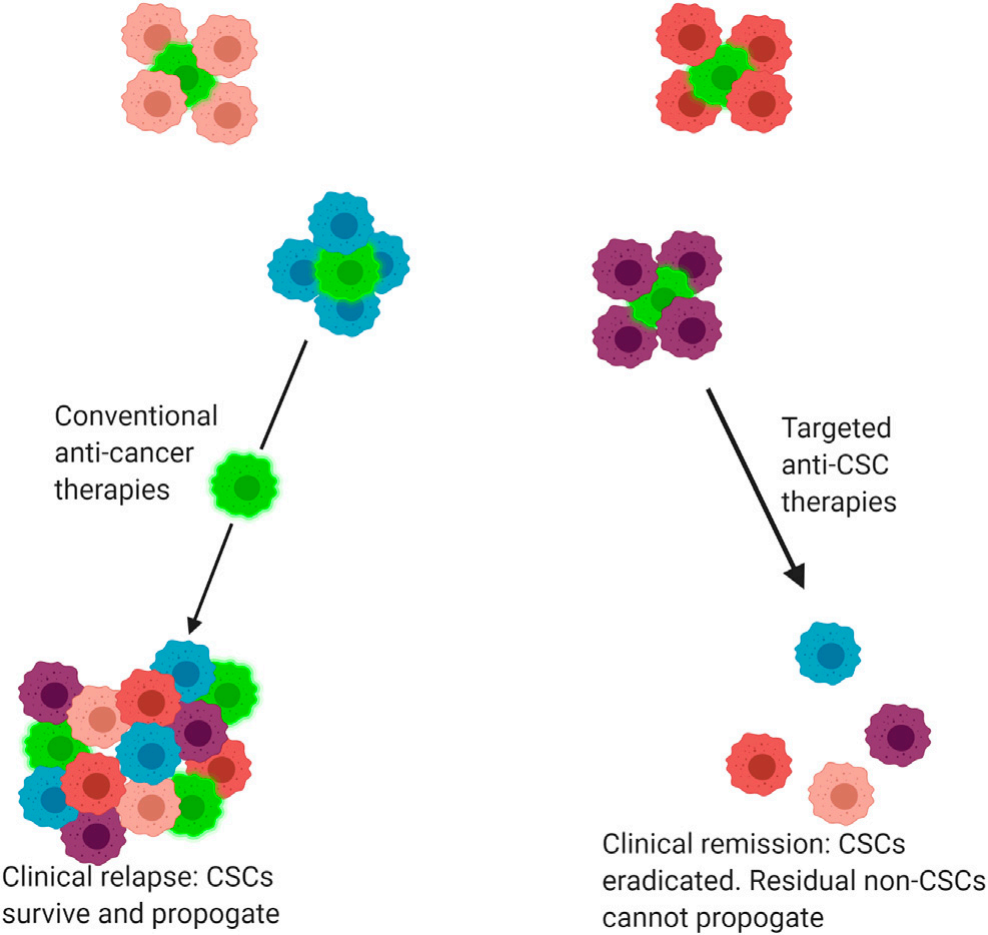
**Response of tumours to conventional and targeted drug therapies**. Tumours are comprised of a heterogenous mix of cancer cells, including both cancer stem cells (CSC) and non-CSC. Conventional anti-cancer therapies primarily target non-CSCs, allowing CSCs to selectively survive and propagate, producing a tumour mass comprised of both CSC and non-CSC subclones. By comparison, some novel precision medicine therapies target CSCs, eradicating the CSC population. The residual non-CSC population have no capacity for self-renewal and thus the tumour regresses or is eradicated, leading to clinical remission. Bright green cells indicate CSCs.

CSCs hold a Darwinian survival advantage over other subclones within a single tumour due to their endogenous resistance against chemo-radiotherapy regimes (Eun et al., 2017; Prieto-Vila et al., 2017). Their ability to generate phenotypically varied clonal populations within a single tumour increases the likelihood of at least one group of tumour cells surviving the assault of anti-cancer treatments (Brooks et al., 2015; Eun et al., 2017). It has been proposed that the limited efficacy of conventional anti-cancer therapies is attributable to the fact that these treatments target the bulk population of non-CSCs within a tumour, allowing small populations of CSCs to persist and propagate, leading to a clinical relapse (Reya et al., 2001; Shibue and Weinberg, 2017), (Figure 2). CSCs are therefore one of the most clinically important contributors to intra-tumour heterogeneity and thus resistance to anti-cancer treatments.

CSCs have recently been shown to possess the ability to dynamically switch between CSC and non-CSC states. This cellular plasticity is regulated by a number of extrinsic and intrinsic factors (Batlle and Clevers, 2017). Extrinsic factors include niches, which are a specialised component of the tumour microenvironment which act to regulate the fate of stem cells via extrinsic signals and cellular interactions, allowing them to interconvert between differentiated and stem-like states (Quail et al., 2012; Cabrera et al., 2015). In addition to this, intrinsic factors at both the genetic and epigenetic level are also implicated, including regulatory transcription factors (TF), DNA methylation and histone modifications (Thankamony et al., 2020). CSCs are regulated by a number of signalling pathways associated with stemness, including Notch, Hedgehog, Wnt/β-Catenin, JAK/STAT, and NF-κB (Chen et al., 2013a). These pathways play a role in the maintenance of stem cell properties and/or regulation of their differentiation through alteration of messenger RNA (mRNA) expression via a specific subset of TFs including OCT3/4, SOX2, c-MYC and Klf-4 (Takahashi and Yamanaka, 2006; Eun et al., 2017). These TFs, amongst others, are thought to act in concert with each other and additional complex molecular processes, including regulatory microRNAs (miRNA), to establish CSC traits in neoplastic cells. The overlapping influences upon CSC plasticity clearly demonstrate the barriers posed to the development of effective anti-cancer drug therapies for GEJA and other malignancies.

### MicroRNAs as Regulators of CSC

miRNAs are a class of small non-coding RNAs which are involved in regulating gene expression through either degradation of their target mRNA or inhibition of mRNA translation, with an overall effect of altered protein expression within cells (Hezova et al., 2016). miRNAs are key in regulating a range of essential biological processes including proliferation, differentiation, survival and apoptosis in many different cell types (Hezova et al., 2016). They have been shown to be aberrantly expressed in various human cancers and play a part in the regulation of CSC characteristics (Khan et al., 2019). In their latter role, they act by targeting many of the mRNAs which are associated with stemness properties (Khan et al., 2019). Certain miRNAs may also contribute to tumorigenesis by regulating the cell cycle components of CSCs to inhibit apoptosis and promote cellular proliferation (Mens and Ghanbari, 2018).

miRNAs involved in CSC regulation include the miR-17-92 family, which regulates the MYC oncogene to protect CSCs against apoptosis; the let-7 family, whose decreased expression is associated with metastasis and chemoresistance; and a wide range of others including miR-21, miR-16 and miR-200 (Li et al., 2014; Mens and Ghanbari, 2018). Although many miRNA families have been shown to regulate organ-specific CSCs, there is considerable overlap between the expression of miRNAs in different solid organ malignancies (Chakraborty et al., 2016). For example, miR-17 is downregulated in OAC and renal cell carcinoma CSCs, yet miR-17 over-expression has been demonstrated in colorectal CSCs (Lichner et al., 2015; Xi et al., 2016). This highlights the molecular complexities of CSC regulation, and thus the difficulties in identifying a suitable targeted therapeutic agent for individual malignancies. Table 3 lists a number of miRNAs known to play a role in regulating gastric and oesophageal CSCs.

**TABLE 3:** List of miRNAs Involved in Regulating Gastric and Oesophageal CSCs.

miRNA ID	Tumour Type	Pattern of Expression	Functional Significance	Reference
miR-15a-3p	Gastric adenocarcinoma	Downregulated	Tumour suppressor	(Wang et al., 2017)
miR-16-1-3p	Gastric adenocarcinoma	Downregulated	Tumour suppressor	(Wang et al., 2017)
miR-17-5p	OAC	Downregulated	Enhanced radiosensitivity	(Lynam-Lennon et al., 2017)
miR-10b	Gastric adenocarcinoma, OAC	Upregulated. Upregulated	Oncomir. Oncomir	(Wang et al., 2015), (Tian et al., 2010)
miR-200a	Gastric adenocarcinoma, OAC	Downregulated, Upregulated	Tumour suppressor. Oncomir	(Chen et al., 2013c)
miR-146b-5p	Gastric adenocarcinoma	Upregulated	Oncomir	(Chen et al., 2013c)
miR-93-5p	Gastric adenocarcinoma	Upregulated	Oncomir	(Li et al., 2018)
miR-219-5p	Gastric adenocarcinoma	Downregulated	Tumour suppressor	(Li et al., 2017)
miR-193-3p	Gastric adenocarcinoma	Downregulated	Tumour suppressor	(Jian et al., 2016)
miR-192	Gastric adenocarcinoma	Downregulated	Tumour suppressor	(Chiang et al., 2012)
miR-215	Gastric adenocarcinoma	Downregulated	Tumour suppressor	(Chiang et al., 2012)
miR-221	OAC	Upregulated	Enhanced chemoresistance	(Wang et al., 2016)

Abbreviations: miRNA, microRNA; miR, microRNA; OAC, oesophageal adenocarcinoma.

### EMT as a Regulator of CSC

**FIGURE 3:**
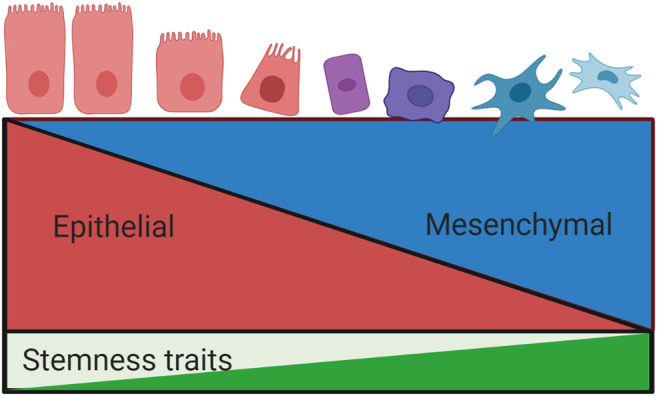
**Relationship between epithelial mesenchymal transition and stemness properties**. The process of epithelial mesenchymal transition involves a loss of epithelial phenotypic traits and a concurrent acquisition of a mesenchymal phenotype. EMT is associated with the development of stemness traits, including invasiveness, metastasis and drug resistance. These processes are tightly regulated by overlapping signalling pathways.

Epithelial mesenchymal transition (EMT) is also believed to play a crucial role in the regulation of CSCs. First described in 1982 by Greenberg and Hay (Greenburg and Hay, 1982), it is a process of lineage transition whereby epithelial cells lose their adhesive properties and acquire a mesenchymal cell phenotype, with corresponding changes in cell morphology and expression of surface markers (Kalluri and Weinberg, 2009). This phenotypic change in neoplastic cells facilitates tumour cell invasion, metastasis and drug resistance (Lamouille et al., 2014; Chen et al., 2017), (Figure 3).

EMT itself is tightly regulated by a wide spectrum of complex cellular signalling pathways. The tumour microenvironment–comprised of a large cohort of stromal cells including cancer associated fibroblasts (CAF), T-lymphocytes, macrophages and myeloid derived suppressor cells–releases a range of cytokines, chemokines and growth factors which act in a paracrine fashion to induce EMT (Kalluri and Weinberg, 2009; Dongre and Weinberg, 2019). These mediators are involved in the activation of a group of EMT-TFs, including the Zeb, Snail, Twist and FOXC families (Medici et al., 2008; Kalluri and Weinberg, 2009; Galvan et al., 2015; Wei et al., 2015; Yu et al., 2015). Once activated, they orchestrate the EMT programme through a series of frequently overlapping intracellular signalling pathways including MAPK, ERK, PI3K, SMADs and Wnt/β-catenin (Tse and Kalluri, 2007; Kalluri and Weinberg, 2009). These pathways are further regulated by multiple intricate cellular interactions involving miRNAs, epigenetic modulators and exogenous inducers (Chen et al., 2017). The TFs, signalling pathways and indeed the regulatory miRNAs which govern EMT have been shown to intersect with those involved in the regulation of CSC characteristics.

An association between EMT and CSC traits was first proposed as an explanation for the ability of tumour cells at the invasive tumour front to metastasize to distant sites (Brabletz, 2012). This relationship has been extensively investigated, with early experimental studies demonstrating an association between EMT and CSC traits in neoplastic cells across a wide range of human carcinomas (Chen et al., 2017). In 2008 Mani *et al* were the first to demonstrate a direct link between EMT and CSCs by inducing EMT in human mammary epithelial cells (HMLE) via ectopic expression of Snail or Twist, or exposure to TGF-β stimulation. Following this, expression of a mesenchymal phenotype and acquisition of stemness traits was witnessed: cells acquired a CD44^high^/CD24^low^ phenotype with the ability to form a mammosphere (Mani et al., 2008). Morel *et al* similarly demonstrated the acquisition of CSC traits in HMLEs following activation of the Ras-MAPK pathway, which is involved in EMT (Morel et al., 2008). However, these in vitro studies induced pure epithelial and mesenchymal states, leading to the assumption that the EMT programme represented a binary switch between phenotypic states, with mesenchymal cells believed to represent CSCs and epithelial cells non-CSCs. Recent evidence now indicates that EMT is best viewed as along continuum, whereby some cancer cells may undergo partial EMT, resulting in a hybrid epithelial/mesenchymal (E/M) phenotype (Grosse-Wilde et al., 2015; Beerling et al., 2016; Nieto et al., 2016; Bierie et al., 2017; Kröger et al., 2019).

This E/M state, rather than the pure mesenchymal phenotype, has recently been shown to correlate with tumour aggressiveness and a poor clinical prognosis (Jolly et al., 2019). Efforts have thus been made to identify the molecular components which promote and regulate this hybrid state, which are referred to as phenotypic stability factors (PSF). Bocci *et al* demonstrated that high expression of nuclear factor erythroid 2-related factor 2 (NRF2) is involved in stabilising the hybrid E/M phenotype, which in turn correlated with poor survival outcomes (Bocci et al., 2019). Additional studies identified GRHL2, OVOL2, NUMB and ΔNp63α as other important PSFs (Watanabe et al., 2014; Dang et al., 2015; Jolly et al., 2016; Bocci et al., 2017). Expression of these factors, in tandem with the EMT-TFs described above, have been shown to facilitate cell migration by preventing cells from undergoing complete EMT. Further interrogation of this model of cellular plasticity is required in order to improve our understanding of cancer progression, metastasis and potentially mechanisms of resistance to anti-cancer drug therapies in GEJA and other malignancies.

Interestingly, both EMT and CSCs are also associated with tumour budding, which is defined as the presence of isolated tumour cells or clusters of up to four tumour cells present in the stroma at the invasive tumour front (Lino-Silva et al., 2018). Tumour buds (TB) are thought to represent the histological correlate of EMT, as they may transiently acquire a mesenchymal phenotype due to activation of the WNT signalling pathway, with associated loss of membranous e-cadherin expression and gain of strong nuclear beta-catenin staining (Zlobec and Lugli, 2010). TBs in colorectal cancer have also been shown to express stem cell markers including LGR5, ALDH1 and CD44, indicating a link between transition to the mesenchymal phenotype and acquisition of stemness traits (Lugli et al., 2020). The presence of TBs has demonstrated utility as a prognostic tool, correlating with risk of disease relapse and death from disease in upper gastrointestinal tumours including OAC and GEJA (Brown et al., 2010; Koelzer et al., 2014; Landau et al., 2014), whilst their potential as a predictive tool remains under investigation. Furthermore, TBs are associated with resistance to conventional anti-cancer therapies, which may be explained by their low proliferative activity and resistance to apoptosis due to up-regulation of anti-apoptotic proteins including RAF-kinase inhibitor protein (RKIP) (Dawson et al., 2014). The presence of these cells in epithelial malignancies, including GEJA, clearly holds potential as a future oncotarget.

## The Role of EMT and CSC in Drug Resistance

Intra-tumour heterogeneity contributes to the efficacy of anti-cancer drug therapies through intrinsic and acquired drug resistance, which develops as a result of both genetic and epigenetic alterations of sub-populations of cancer cells within the tumour mass (Esteller, 2008; Shibue and Weinberg, 2017). The relative sensitivities of isolated CSC-enriched tumour sub-populations to chemotherapy, radiotherapy, immunotherapy and molecularly targeted therapies have been extensively investigated, with analyses demonstrating a far greater survival of CSCs compared to non-CSCs across all treatment modalities and across multiple different cancer types (Graham et al., 2002; Levina et al., 2008; Dallas et al., 2009; Shibue and Weinberg, 2017).

EMT activation confers resistance to many different types of therapeutic agents through a range of mechanisms, including elevated expression of anti-apoptotic proteins such as Bcl-XL; slow stem cell proliferation rates and increased levels of ATP-binding cassette (ABC) transporters that mediate drug reflux (Singh and Settleman, 2010; Shibue and Weinberg, 2017). For example, Snail and Slug confer resistance to chemotherapy in many cancers through antagonization of p53-mediated apoptosis and by regulation of other genes involved in cell death (Dongre and Weinberg, 2019). The miR-200 family play a contributory role in treatment resistance, restoring chemosensitivity in aggressive cancer cells through reversal of EMT (Cochrane et al., 2010). This association is further corroborated by studies which demonstrated a strong link between treatment resistance and the altered expression of genes associated with EMT in cancer cells (Farmer et al., 2009; Byers et al., 2013).

Early results from clinical trials indicate that CSCs play a key role in regulation of resistance to anti-cancer drugs. A phase II clinical trial of patients with gastric cancer showed that patients who received chemotherapy with Vismodegib–a hedgehog inhibitor–held a survival advantage if their tumour had high expression of CSC marker CD44 (Yoon et al., 2014). The use of immunotherapy approaches to target CSCs are also under investigation, focusing on therapies which target the CSC traits of immune resistance and immunosuppression (Codd et al., 2018). Despite these early advances, a greater understanding of the relationship between EMT, CSCs and their mechanisms of drug resistance would undoubtedly enhance drug development and clinical outcomes for patients.

### CSC Markers in GEJA

Therapeutic targeting of CSCs is limited by difficulties in characterization of appropriate CSCs across many solid and haematological malignancies. A range of markers have been recognised for identification of CSCs, including cell surface markers CD133, CD44, CD24 and CD66 and ALDH1A1 (Prasetyanti and Medema, 2017). Unsurprisingly, given their shared characteristics, the markers used to isolate CSCs overlap greatly with those used in the identification of normal adult stem cells in non-neoplastic tissues (Brungs et al., 2016). Their clinical utility is somewhat hampered by the fact that expression of CSC markers is not uniform across different malignancies: heterogenous expression may be observed within a single tumour, between cancer subtypes and even between patients within the same tumour subtype (Visvader and Lindeman, 2012). Furthermore, the inherent plasticity in the process of acquisition of CSC traits further complicates the isolation of CSCs for further study.

Several studies exist within the literature regarding the identification, regulation and clinicopathologic characteristics of CSCs and CSC-like cells in both gastric and oesophageal cancers, amongst a wide range of other malignancies. Whilst studies pertaining specifically to CSCs in GEJA are sparse, it must be remembered that studies investigating the role of CSCs in both OAC and gastric cardia adenocarcinomas will include a proportion of GEJAs. Here we describe some of the most common CSC markers used in gastric and oesophageal malignancies.

#### CD133

CD133, also known as Prolamin-1, is a five transmembrane glycoprotein plasma membrane protein that has been used to identify putative CSCs in a range of tumours including colon, pancreas, prostate, stomach and oesophagus (Brungs et al., 2016). It plays a role in regulation of the lipid component of the plasma membrane, yet its precise function remains unknown (Codd et al., 2018). Whilst frequently used as a marker of CSCs, CD133 is not a CSC-specific antigen as it is also expressed in a number of differentiated epithelial cells in various organs (Wu and Wu, 2009). The use of different CD133 clones complicates comparisons between studies, leading to poor reducibility and potential for erroneous results (Hermansen et al., 2011). Despite this, an early study investigating the utility of CD133 as a target for anti-CSC therapies in ovarian cancer has shown promising results (Skubitz et al., 2013).

A meta-analysis investigated the correlation between CD133^+^ gastric cancers and clinical outcomes in 773 patients, identifying worse accumulative 5 year OS rates in CD133^+^ patients (21.4%) as compared with CD133^−^ patients (55.7%), in addition to a close correlation between CD133 over-expression and adverse clinicopathological features (Wen et al., 2013). A more recent study demonstrated higher levels of CD133^+^ cells in blood samples from gastric cancer patients, which correlated with poor prognosis, as compared to unmatched normal controls (Xia et al., 2015).

The role of CD133 in drug resistance has been described through analysis of the ability of SP1049C—a pluronic-based micellar formulation of Doxorubicin that has demonstrated safety and efficacy in patients with advanced OAC and GEJA in a phase II trial–to deplete CD133^+^ CSCs and decrease cancer cell tumorigenicity *in vivo* (Alakhova et al., 2013). These findings suggest a link between CD133^+^ CSCs and drug resistance in OAC.

#### CD44

CD44 is a transmembrane glycoprotein that is expressed on both CSCs and differentiated adult cells, including endothelial cells and hepatocytes, thus it cannot be regarded as a CSC-specific antigen. It has a wide range of physiological roles including adhesion, migration, differentiation, growth and survival (Ponta et al., 2003). It serves as a putative CSC marker in a range of malignancies including colon, brain, stomach and oesophagus (Brungs et al., 2016). CD44 is encoded by the 20 exon CD44 gene, which is subject to alternative splicing (Lau et al., 2014). It has been proposed that CD44 variants (CD44v) are more specific in their identification of cells with tumorigenic potential when compared to the standard isoform (CD44s) (Thapa and Wilson, 2016). A number of studies have identified CD44v in metastatic deposits from a range of solid organ malignancies, which were associated with a poorer prognosis (Mulder et al., 1994; Kaufmann et al., 1995; Ni et al., 2014; Ozawa et al., 2014). Specific CD44 isoforms have been identified as potential targets for anti-cancer therapies: early studies are investigating the potential for therapeutic targeting of CD44^+^ CSCs in breast cancer (Aires et al., 2016).

CD44v6 expression in gastric cancer resection specimens is associated with poorer clinical outcomes including distant metastasis, lymph node metastasis and depth of invasion (Liu et al., 2005; Chen et al., 2013b). CD44^+^ circulating tumour cells (CTCs) in patients with gastric cancer were also shown to correlate with the clinicopathologic characteristics of the resected tumour specimens, including disease stage and venous invasion, whilst CD44^−^ CTCs did not (Watanabe et al., 2017). The association between loss of CD44 expression and poor survival outcomes in patients with OAC has also been described (Honing et al., 2014). These findings suggest that CD44 is useful as a putative CSC marker and a predictor of patient outcomes in gastric adenocarcinoma and OAC.

#### ALDH1

Within the human genome, the aldehyde dehydrogenase (ALDH) family comprises a reported 19 functional genes which encode enzymes involved in the oxidative metabolization of endogenous and exogenous aldehyde substrates, including lipids and amino acids (Tomita et al., 2016). ALDH1 has 3 isoforms (ALDH1A1, ALDH1A2 and ALD1A3) and is a marker of both stem cells and CSCs, with expression observed in colon, pancreas, breast and prostate cancers (Brungs et al., 2016; Tomita et al., 2016). ALDH has been shown to attenuate oxidative stress: CSCs contain lower levels of reactive oxygen species (ROS) than differentiated tumour cells, allowing them to survive under conditions of metabolic and oxidative stress (Vassalli, 2019). The ALDH family is in fact a target of the TF NRF2, which is known to promote the hybrid E/M phenotype and thus tumorigenic properties, through its antioxidant defences (Luo et al., 2018).

Katsuno *et al* demonstrated CSC properties of self-renewal and increased tumorigenicity in isolated ALDH1^+^ cells from gastric cancer cell lines (Katsuno et al., 2012). High ALDH expression has also been correlated with poor clinical outcomes in pancreatic, ovarian and prostate cancers (Kuroda et al., 2013; Le Magnen et al., 2013; Fitzgerald and Mccubrey, 2014). Furthermore, acquired drug resistance in tumour cells is associated with transcriptional activation of ALDH1 expression (Yoshida et al., 1993). Early studies have investigated the utility of therapies targeting ALDH1 positive CSCs in breast, ovary and NSCLC (Li et al., 2008; Duan et al., 2014; Schech et al., 2015; Wu et al., 2015; MacDonagh et al., 2017). A phase II trial investigated the effect of administering Disulfiram–a potent ALDH inhibitor–in addition to standard chemotherapy to patients with NSCLC, demonstrating good drug tolerance and a prolonged survival (Nechushtan et al., 2015). Thus, ALDH1 holds great potential as a CSC target for novel drug therapies.

ALDH isoforms ALDH1A3 and ALDH1L1 have shown potential as prognostic markers and therapeutic targets in gastric cancer (Li et al., 2016), whilst Ajani *et al* showed that ALDH1^+^ tumour cells from OAC and GEJA resection specimens were more resistant to chemoradiotherapy, as compared to tumour cells with low ALDH1 expression (Ajani et al., 2014; Honing et al., 2014). Brungs *et al* examined the significance of the expression of CD133, CD44 and ALDH1 in metastatic deposits of GEJA: CD44 and ALDH1 expression were both significantly associated with poorer OS, and CD44 positivity was identified as an independent prognostic marker (Brungs et al., 2019).

#### EpCAM

The epithelial molecular adhesion molecule (EpCAM) is a transmembrane glycoprotein present in most epithelial tissues that plays a role in cell adhesion, migration and differentiation (Imano et al., 2013). EpCAM is commonly expressed in gastric cancer, with one study demonstrating CSC characteristics within the EpCAM^+^ tumour population, but not in EpCAM^-^ tumour cells (Wenqi et al., 2009). Imano *et al* showed that peritoneal metastases of gastric cancer express higher levels of EpCAM, as compared with biopsy samples of the primary tumour, indicating that only gastric cancer cells with high EpCAM expression may metastasize to the peritoneum (Imano et al., 2013). Despite this, most gastric cancers are EpCAM^+^, thus it must be used in conjunction with other more specific markers in identification of gastric CSCs (Brungs et al., 2016). Sun *et al* demonstrated that resistance to treatment with Adriamycin, Cisplatin and 5-FU (ACF) was associated with an increase in EpCAM and CD90 expression in OAC, suggesting a role for these putative CSC markers in establishing drug resistance (Sun et al., 2018).

#### miRNAs

A number of miRNAs have been linked to the expression of gastric CSCs. miR-196a-5p has been shown to be upregulated in CD44^+^ gastric CSCs, and to play a key role in EMT and invasion through targeting of the Smad4 signalling pathway (Pan et al., 2017). High miR-501-5p levels were associated with poor OS and were shown to induce a CSC-like phenotype in gastric cell lines through activation of Wnt/β-catenin signalling pathways (Fan et al., 2016). Upregulation of miR-132 in gastric CSCs was linked to chemoresistance (Zhang et al., 2017). These miRNAs hold great promise as a targetable molecule in the treatment of gastric cancer, yet extensive work is required to validate their prognostic significance and mechanisms of action.

miRNAs have also been implicated in the regulation of CSC traits in OAC tumour cells. Downregulation of miR-17-5p in OAC tumour cells with CSC traits was shown to produce a radioresistant phenotype (Lynam-Lennon et al., 2017). Similarly, over-expression of miR-221 in OAC was associated with resistance to 5-FU based chemotherapeutic regimens; experimental knockdown in resistant cells resulted in dysregulation of CD44 in addition to other Wnt/β-catenin signalling target genes (Wang et al., 2016). These findings, taken in conjunction with protein and potential mRNA CSC markers, merit greater interrogation as the co-expression of different molecular markers may hold great promise as targets for anti-cancer therapies.

## Discussion

**FIGURE 4:**
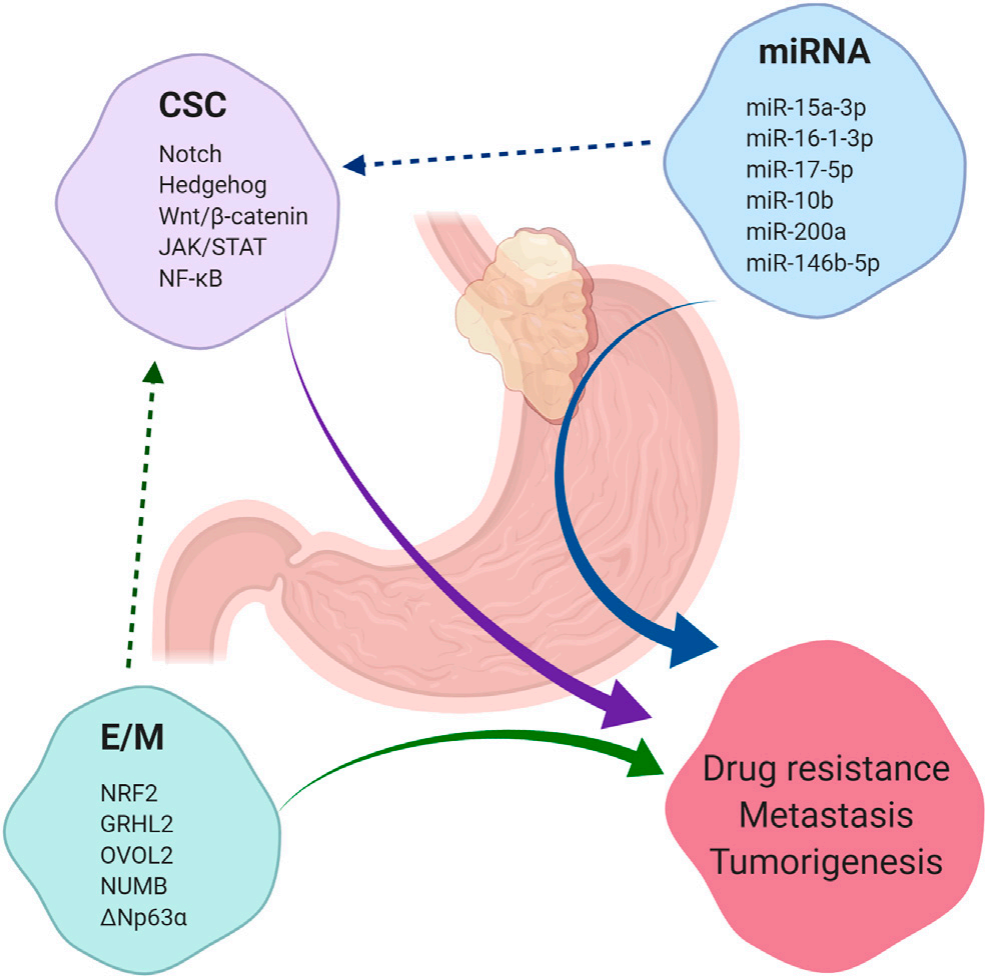
**Summary of factors which contribute to tumorigenesis, drug resistance and metastasis**. Cancer stem cells (CSC), microRNAs (miRNA) and the epithelial/mesenchymal (E/M) phenotype each contribute to the development of tumorigenesis, drug resistance and metastasis across a range of malignancies, including gastroesophageal junction adenocarcinoma (GEJA). E/M and certain miRNAs have been shown to regulate the acquisition of stemness properties in cancer cells. A selection of regulatory pathways which govern CSC regulation are listed, in addition to a selection of miRNAs shown to play a role in regulation of CSCs in gastric and oesophageal adenocarcinomas. A selection of phenotypic stability factors which regulate the E/M hybrid state are also listed.

GEJAs are associated with poor clinical outcomes and high rates of drug resistance. CSCs present a novel therapeutic target in GEJA, yet our knowledge of markers of putative GEJA CSCs and their regulatory pathways has been largely extrapolated from studies looking at gastric and oesophageal CSCs (Figure 4). Thus, our understanding of the mechanisms regulating the acquisition of stemness traits in GEJA neoplastic cells remains incomplete. In light of the growing opinion that GEJ tumours are best regarded as a disease entity in their own right, more focused attention is required to determine the specific molecular characteristics of GEJA.

The future directions for research into CSCs in GEJA are clear. An improved understanding of the phenotype of CSCs in GEJA, as distinct from non-CSCs, is required to guide targeted drug development. It is also important to accurately characterise the differences in molecular biology of both primary tumours and metastatic deposits, as potential variations may render targeted therapies useful in different disease settings. Furthermore, an enhanced knowledge of the regulatory pathways and miRNAs governing CSCs in GEJA would both facilitate drug development programmes and improve clinical prognostication, thus helping to provide the best possible treatment for this patient population.
